# A Comparative Analysis of Biomarker Expression and Molecular Subtypes of Pure Ductal Carcinoma In Situ and Invasive Breast Carcinoma by Image Analysis: Relationship of the Subtypes with Histologic Grade, Ki67, p53 Overexpression, and DNA Ploidy

**DOI:** 10.4061/2011/217060

**Published:** 2011-08-17

**Authors:** Venetia R. Sarode, Jeong S. Han, Danielle H. Morris, Yan Peng, Roshni Rao

**Affiliations:** Departments of Pathology and Surgical Oncology, The University of Texas Southwestern Medical Center, 5323 Harry Hines Boulevard, Dallas, TX 75390-9073, USA

## Abstract

There is a paucity of data regarding molecular subtypes of pure ductal carcinoma in situ (pDCIS). We evaluated the expression of ER, PR, HER2, Ki67, and p53 and DNA ploidy in 118 pDCIS and 100 invasive breast carcinomas (IBCAs) by routine IHC and classified them according to molecular subtypes. Quantification of biomarkers and DNA ploidy was performed by image analysis. Expression of ER, PR, and high ki67 was more frequent in pDCIS compared to IBCA. High-grade tumors had lower ER and PR expression, high Ki67, overexpression of HER2 and p53, and DNA aneuploidy. Luminal A and HER2 subtypes were more common in pDCIS, and triple negative was more prevalent in IBCA. In both groups, HER2 and triple negative subtypes were characterized by high ki67, overexpression of p53, and DNA aneuploidy compared to luminal subtypes. Molecular subtypes of IBCA are distinct from those of pDCIS. Invasion is characterized by change in phenotype in some tumors.

## 1. Introduction

Ductal carcinoma in situ (DCIS) is a complex disease with diverse clinical presentation, histologic subtypes, and biologic behavior [1]. Although DCIS is considered a direct precursor of invasive carcinoma, the rate of progression is highly variable with some types of DCIS progressing faster than others. The conventional parameters such as histologic patterns, nuclear grade, and presence of necrosis are used for grading and determining prognosis in DCIS [1–3]. However, morphologic features alone do not reflect the true biology of this disease. The identification of markers that can predict recurrence and/or invasion in DCIS is critical. Some studies have showed that expression of ER, PR, HER2, Ki67, and p53 correlate with tumor grade rather than invasion [1–5].

Gene expression analysis has demonstrated several molecular subtypes of IBCA; these include the luminal A, luminal B, HER2, and triple negative/basal subtypes [6, 7]. Recent studies have shown that expression of ER, PR, and HER2 by IHC can be used as a surrogate tool for the molecular subtyping of IBCA [8, 9]. Proliferation is an important component in the molecular classification of IBCA [8, 10, 11]. The MKi67 gene encodes the Ki67 protein, a robust marker of cell proliferation and a predictor of poor outcome [11].

While the molecular subtypes of IBCA have been well characterized, far less is known about the biologic subtypes of pDCIS. A detailed analysis of biomarker expression in pDCIS has not been well documented, and there are very few studies that have compared biomarker expression and molecular subtypes in pDCIS with IBCA. There is a paucity of data regarding the relationship of the molecular subtypes with tumor grade, Ki67 and p53 expression, and DNA ploidy [9, 12]. 

In the present study, we performed a comparative analysis of biomarkers (ER, PR, HER2, Ki67, and p53) expression and DNA ploidy in pDCIS and IBCA by automated image analysis using whole tumor sections and determined if there were quantitative differences in their expression in the two groups. We also attempt to classify pDCIS and IBCA according to molecular subtypes using IHC as proxy for gene expression and to determine if there were any differences in the prevalence of the subtypes in the two groups. We also investigated the relationship of Ki67 index, tumor grade, DNA ploidy, and p53 overexpression with molecular subtypes of pDCIS and IBCA.

## 2. Materials and Methods

This retrospective study consists of 118 consecutive patients with a diagnosis of pDCIS and 100 consecutive cases of IBCA on final surgical excision. Patient demographics, tumor size, and types of surgery were extracted from the electronic medical records of UT Southwestern Medical Center and Parkland Health and Hospital Systems after approval by the Institutional Review Board. All pathology data was obtained from electronic laboratory information system of the Department of Pathology. Grading of DCIS and IBCA was performed using the World Health Organization (WHO) criteria into low (grade 1), intermediate (grade 2), and high (grade 3). Size of DCIS was determined by the extent of the lesion in consecutive sections or by the number of involved sections and gross measurement. The architectural patterns of DCIS were also recorded, and comedo necrosis was evaluated as a separate parameter but was not quantitated. Grading of IBCA was done using the modified Bloom-Richardson Nottingham scoring system. 

Biomarker expression and DNA ploidy were prospectively performed as part of the patient's clinical workup. As a matter of routine, the most representative tumor section was selected for biomarker analysis. In patients with IBCA, paraffin block with predominantly invasive tumor was selected and image analysis was performed only on the invasive tumor, regardless of the presence or absence of in situ component. 

### 2.1. Immunohistochemistry (IHC) and Image Quantitation of Biomarker Expression

Slides were stained on an automated immunostainer (Dako autostainer, Carpentaria, CA). Monoclonal antibodies from the Dako Medical Systems, Carpenteria, CA were used for ER (clone 1D5, 1 : 800), PR (PgR 636, 1 : 1000) and Ki67 (MIB-1, 1 : 300), HER2/neu (1 : 600), and p53 (DO-7, 1 : 2200, Dako, Carpenteria, Calif, USA). Scoring and quantification of ER, PR, Her2/neu, p53, and ki67 was performed on the most representative area of the tumor using the computerized Automated Cellular Imaging System (ACIS, Clarient, Inc. San Juan Capistrano, Calif, USA). The ACIS system consisted of an automated robotic bright-field microscope module, a computer, and a Windows-NT-based software interface. Subregions were selected from the digital images of the IHC-stained slides for analysis. Positive staining in 5% or more of the tumor cells for ER and PR in 10 selected subregions of the tumor was scored as positive for ER and PR expression. The results of ER and PR were reported as percent of positive staining nuclei, and staining intensity was graded from 1+ to 3+. To assess HER-2 overexpression, ACIS provided an average score for 5 selected subregions of the tumor with the highest cytoplasmic membrane staining intensity for HER-2. Tumors with more than 10% cells with an average score ≥2.0 were considered to have HER-2 overexpression, this was equivalent to a 3+ positive staining by manual scoring. Scores between 1.4 and 1.9 were reported as borderline (or 2+ by manual scoring), and <1.4 were reported negative. Computer-generated results were confirmed by manual review by pathologists with experience in image analysis. Tumors with ER and PR scores of less than 5% were considered negative expression. All cases with positive or borderline HER-2 results on IHC were confirmed by fluorescent in situ hybridization (FISH) using the FDA approved PathVysion kit (Abbott-Vysis Lab Abbott Park, Ill, USA) according to the manufacturer's protocol. Briefly, dual-color FISH was performed with the HER2 probe labeled with spectrum red and chromosome 17 specific centromere (D17Z1) probe labeled with spectrum green on deparaffinized tumor sections cut from the same block. Fluorescent signals in at least 60 nonoverlapping interphase nuclei with intact morphology were scored with ×100 objective, using a fluorescence microscope. Only tumor cells from the area designated on the H&E slide by the pathologist were scored for the number of red (HER-2) and green (chromosome 17) signals. A ratio of the number of fluorescent signals of HER-2 to chromosome 17 greater than 2.0 was reported as HER2 amplified. 

The Ki67 index of >10% was considered to be significant. P53 expression in >10% of the tumor cells were considered as overexpression. Bcl-2 immunostaining was performed in the pDCIS cases but not on the invasive group.

### 2.2. DNA Ploidy Analysis

DNA analysis was performed by image analysis using Feulgen DNA stains on paraffin sections from the same tumor block that was used for biomarker analysis. DNA indices and ploidy were analyzed using the Autocyte Pathology Workstation (Tripath, Burlington, NC, USA). Briefly, a total of 200–300 nuclei were collected was and mean DNA index reported for each patient. DNA index was obtained by measuring the optical density of tumor cells in comparison with those of the nonneoplastic stromal cells in the sample using the latter as the diploid reference (value of 1.0). Tumors were classified into diploid and aneuploid/multiploid based on the DNA indices.

### 2.3. Molecular Classification of pDCIS and IBCA Using IHC

Pure DCIS and IBCA were classified according to the molecular subtypes. We used the Ki67 score of 14% as the cut-off for distinguishing luminal A (ER+, PR±, HER2−, Ki67 <14%) and luminal B (ER+, PR±, HER2−, Ki67 ≥14%) subtypes; luminal-Her2 (ER+, PR±, HER2+); HER2+ (ER−/PR−/HER2+) and triple negative (ER−/PR−/HER2−). The relationship of the molecular subtypes with tumor grade, Ki67 index, p53, and DNA ploidy was analyzed. Quantitative expression of ER, PR, HER2, Ki67, and p53 by image analysis was correlated with tumor grade in both the pDCIS and IBCA groups. We also compared tumor grade and ploidy with luminal (luminal A, B, lum-HER2) versus nonluminal (HER2 and triple negative) types. 

Statistical analysis was performed by one-way ANOVA test followed by Tukey post hoc for comparison of continuous data and Fisher's exact test and Chi-square for categorical data. The two-way ANOVA with Bonferroni post hoc test was used for comparing Ki67 in the different subtypes of pDCIS and IBCA using the Prism 5 software (Graphpad Software Inc, San Diego, Calif, USA).

## 3. Results

### 3.1. Comparison of Biomarker Expression in pDCIS versus IBCA

The mean age, tumor size, and grade were similar in the two groups (Table 1). The frequency of expression of different biomarkers in the two groups is summarized in Table 2. In pDCIS, ER, and PR expression was significantly higher as compared to IBCA (*P* = 0.007 and *P* = 0.005, resp.). Although HER2 overexpression was higher in pDCIS compared to IBCA, the difference was not statistically significant (*P* = 0.285). Ki67 scores of >10% was significantly higher in IBCA compared to pDCIS group (*P* = 0.003). Overexpression of p53 (>10%) was more frequent in IBCA; however, the difference was not statistically significant.

### 3.2. Comparison of Biomarker Expression and DNA Ploidy with Histologic Grade of pDCIS (Table 3)

Expression levels of ER and PR decreased significantly with increasing tumor grade (*P* = 0.003 and *P* = 0.004, resp.). HER2 overexpression was absent in low-grade DCIS. It was positive in 6 of 46 (13%) of intermediate grade and 28 of 63 (44%) of high-grade DCIS (*P* = 0.0007). The mean HER2 gene copy number by FISH was 5.89 in high grade compared to 4.47 in the intermediate-grade group, *P* = 0.30. Comedo necrosis was present in 75% (27/36) of HER2 positive cases compared to 67% (55/82) in HER2 negative (67.0%), *P* = 0.51. The Ki67 scores increased significantly with higher grades of DCIS (*P* = 0.0002). Bcl-2 expression decreased significantly with increasing DCIS grade (*P* = 0.001). Overexpression of p53 was also more frequent in high-grade Pdcis, compared to low grade (*P* = 0.002).

Tumor DNA content was diploid in 38/55 (69%) of grade 1-2 pDCIS and 47/63 (74.6%) of grade 3 pDCIS were aneuploid (*P* < 0.0001).

### 3.3. Comparison of Histologic Grades of IBCA with Biomarker Expression and DNA Ploidy (Table 4)

In the IBCA group, ER and PR scores decreased significantly with increasing tumor grade (*P* < 0.0001 and *P* = 0.005, resp.). The mean HER2 gene amplification ratios by FISH were 3.3, 5.2, and 4.3 in grades 1, 2, and 3 IBCA respectively, and this was not statistically significant. Grade 3 IBCA showed significantly higher p53 overexpression compared to grade 1 and 2 IBCA (*P* = 0.018). The Ki67 index increased significantly with tumor grade (*P* < 0.0001). Grade 1 and 2 IBCA, 29/34 (85.2%) had diploid DNA, and 35/66 (53.0%) of grade 3 tumors were aneuploid (*P* = 0.0002).

### 3.4. Comparison of Molecular Subtypes of pDCIS and IBCA by IHC (Table 5)

The prevalence of the subtypes differed significantly in the two groups. Luminal A was more common in pDCIS compared to IBCA (*P* = 0.011), and triple negative subtype was higher in IBCA (*P* < 0.0001). HER2 subtype was higher in pDCIS than IBCA, but this was not statistically significant (*P* = 0.225). 

 The luminal versus nonluminal pDCIS and IBCA showed significant association with tumor grade. In the pDCIS group, luminal tumors were frequently of lower grade (grade 1 and 2), 51/97 (52%), and nonluminal tumors were more likely to be grade 3, 17/21 (80.9%), *P* = 0.007. Similarly, in the IBCA group, luminal tumors were predominantly of lower grade, 48/63 (76.1%), and nonluminal were frequently of high grade, 23/33 (69.6%), *P* < 0.0001. 

The pDCIS molecular subtypes showed significant association with DNA ploidy; luminal tumors were more likely to have diploid DNA, 53/97 (54.6%), and non luminal tumors were frequently aneuploid, 20/21 (95.2%), *P* < 0.0001. Similar association was seen in the IBCA group; luminal tumors were mostly diploid, 30/66 (45.4%), and non luminal subtypes were frequently aneuploid/multiploid, 28/33 (84.8%), *P* = 0.0035. 

A comparison of Ki67 index and p53 overexpression with the different subtypes is shown in Table 6. Ki67 index increased significantly in luminal B, lum-HER2, HER2, and triple negative in pDCIS (*P* < 0.0001) and IBCA (*P* < 0.0001). A significant association with p53 overexpression was also noted for both pDCIS (*P* = 0.0014) and IBCA (*P* < 0.0001). The lum-HER2, HER2, and triple negative subtypes of invasive tumors had significantly higher Ki67 proliferation indices compared to similar subtypes of pDCIS (two-way ANOVA and Bonferroni post hoc test (*P* < 0.01), Table 7).

## 4. Discussion

In the current study, biomarker expression was performed prospectively on whole tumor sections at the time of the patient's diagnosis. Previous studies on biomarker expression were performed retrospectively on tissue microarray obtained from archived tumor blocks [9, 13]. We used automated image analysis for quantification of biomarker expression. The advantage of this technique is the consistency of results with less intra- and interobserver variability compared to manual estimation [14–16]. Several studies have shown excellent correlation between manual and automated analysis of biomarker expression in breast cancer [14–16]. In our study, semiquantitative analysis of ER and PR levels demonstrated significant inverse relationship with tumor grade in both pDCIS and IBCA. Previous studies have shown that ER and PR status is inversely correlated with tumor grade; however, quantification was not performed [1–4, 13, 17]. Furthermore, we have shown that the expression of ER and PR in pDCIS was significantly higher compared to IBCA. This is in contrast to previous studies where the expression was found to be higher in IBCA than DCIS using tissue microarray [4, 9]. Unlike whole tumor sections, tissue microarray may lead to sampling error especially in tumors with heterogeneous expression [18]. Also the cutoff limit for defining positive expression may produce different results depending on the positive threshold used in the study. Higher tumor grade was also associated with HER2 overexpression in both pDCIS and IBCA consistent with previous findings [2, 4, 9, 12, 13, 17].

There are very few studies that have compared molecular subtypes of pDCIS and IBCA [9, 17]. In the present study, we have demonstrated that the prevalence of the subtypes differed significantly in the two groups. In pDCIS, the luminal A was the most common (37%) followed by HER2 (31%) and luminal B (26%); the triple negative subtype was the least common (6%). In contrast, in the IBCA group, luminal B was the most common (30%) followed by triple negative (26%) and HER2 subtypes (23%). In both groups, luminal A tumors were predominantly of low nuclear grade, with low ki67 index, negative p53, and HER2 overexpression. Additionally, these tumors were frequently diploid compared to nonluminal tumors. Luminal A tumors originate from differentiated luminal progenitor cells characterized by high ER/PR and Bcl-2 expression [19]. The significantly higher prevalence of luminal A in pDCIS compared to IBCA may be partly attributed to an increase in screening mammography which may have resulted in higher rates of detection of these very early lesions. The incidence of pDCIS has increased significantly over the past decade due to screening mammography [20]. Another possibility is that some of the low-grade (luminal A) DCIS may not progress to IBCA during the patient's lifetime due to their low growth fraction [21].

The prevalence of luminal B subtype was similar in both pDCIS and IBCA. Tamimi et al. showed that luminal B subtype was more frequent in pDCIS compared to IBCA [9]. In their study, the classification of luminal tumors was based on the criteria used in Carolina Breast Cancer Study [22] in which luminal B tumors were defined by the coexpression of ER and HER2. In our study, we used Cheang et al.'s criteria [11] for classifying luminal tumors, which also takes into account the Ki67 index besides ER, PR, and HER2 expression. According to their study, the best Ki67 cutoff value for differentiating luminal A from B tumors was 14%. It has been shown that the MK167 gene that encodes the Ki67 protein was highly expressed in luminal B tumors [6, 7, 10, 11]. Luminal B tumors that coexpressed HER2 were classified as luminal-HER2. This subtype comprised only 30% of the luminal B tumors [11]. Some authors believe that luminal B tumors are negative for HER2 [23]. 

In the current study, there was a trend towards higher prevalence of HER2-positive pDCIS (31%) compared to IBCA (23%). Several studies have demonstrated significantly higher HER2 overexpression in pDCIS compared to IBCA [4, 9, 12, 13, 17]. However, in most of these studies, testing for HER2 was performed by IHC only and the criteria for HER2 overexpression were much lower compared to standard guidelines. In our study, tumors with HER2 scores of 2+ and 3+ by IHC were also confirmed by FISH analysis and only HER2-amplified tumors were considered positive. Since FISH is the gold standard for HER2 testing, we believe that our findings reflect the true prevalence of HER2 in our study population. Studies of HER2 amplification by FISH have also shown higher amplification in pDCIS compared to IBCA [24, 25].

The clinical significance of HER2 overexpression in DCIS is not known at this time. Some suggests that HER2 overexpression in DCIS predicted a more rapid progression to invasive carcinoma [26, 27]. It has been suggested that HER2 promotes the expression of factors that enhances invasion [26, 27]. Others theorized that HER2 expression might be upregulated in the early stages of invasion and downregulated again in the invasive stage of the tumor [26]. Clonal evolution may also give rise to HER2 negative clones that develop invasive capabilities [17]. The increased rate of detection of high-grade DCIS by screening mammography may also contribute to the higher prevalence of HER2-positive pDCIS [28]. 

Although we did not examine the expression of basal markers in the triple negative tumors, the majority of them have been shown to have basal phenotypes [29]. The prevalence of basal subtype pDCIS was 8% in a population-based study by Livasy et al. compared to 20% in IBCA [12, 22]. Others have also shown similar findings [13]. It has been suggested that basal-like pDCIS may have a short in situ phase [12]. This may explain their lower prevalence and the difficulty in identifying DCIS component in basal IBCA. Dabbs et al. demonstrated that the DCIS component in basal like IBCA was very focal and comprised a small percentage (<10%) of the entire tumor [30]. 

The lower ER, PR and HER2 expression in some IBCA compared to pDCIS as shown in this study may explain the higher prevalence of triple negative tumors in the former. This may support the theory that triple negative IBCA may be an acquired phenotype that evolved from either a luminal B or HER2 subtype of DCIS [12]. It is also plausible that tumor progression may be associated with emergence of clones that have molecular signatures different from the cell of origin [17]. The progression to steroid and HER2 independence in some IBCA may also be due to the ability of some tumors to constitutionally express autocrine growth factors making them less hormone or HER2 dependent. We did not analyze basal marker expression in our triple negative tumors; it is conceivable that some may have expression profiles that are different from the true basal phenotype. It is well known that triple negative tumors are a heterogeneous group with diverse morphologic and molecular profiles [29].

 Ki67 index has been shown to have prognostic significance in breast cancer [8, 11]. However, there is lack of uniformity in reporting Ki67 values due to different techniques and cut off values [11]. Moreover, intratumoral heterogeneity in proliferation is another reason for the variability in the Ki67 index [31]. Quantitative assessment of Ki67 expression by image analysis can provide accurate estimation of the proliferation index. We found that IBCA had significantly higher proliferation compared to pDCIS. Ma et al. [32] showed significantly higher Ki67 in invasive ductal carcinoma compared to those associated with DCIS. 

The highly significant association of Ki67 index and molecular subtypes of pDCIS with IBCA has not been shown previously. Proliferation was highest in the triple negative tumors followed by HER2 and luminal B subtypes. Luminal A had the lowest proliferation (<10%). Others have shown only a modest increase in Ki67 index using tissue microarray [8]. However, tissue microarray may lead to underestimation of proliferation, which can vary in different areas of the tumor [31]. 

The differences in proliferative activity among the molecular subtypes of invasive carcinoma have been shown in gene microarray studies [6, 10, 11]. We have demonstrated for the first time the relationship between proliferation and molecular subtypes of both pDCIS and IBCA using Ki67 expression by routine IHC. Additionally, the Ki67 indices in the luminal-HER2, HER2, and triple negative subtypes of IBCA were significantly higher compared to similar subtypes of pDCIS. 

The association between increased proliferation and DCIS progression has been elucidated by Ma et al., where genes involved in cell proliferation and DNA repair were expressed at higher level in high-grade DCIS, which were further elevated in IBCA revealing a link between proliferation, tumor grade, and invasion [32]. Additionally, stromal factors may play a critical role in influencing tumor growth. It is known that tumor cells can modify the stromal environment to produce bioactive factors that enhance proliferation, survival, and invasion [33, 34]. 

One limitation of this study is that we did not analyze the DCIS component of the IBCA for comparison with pDCIS since this is a retrospective analysis. Several studies have shown no differences in the biomarker expression in the DCIS component of IBCA versus IBCA only [17, 35]. We did not include basal markers for further characterization of the triple negative tumors. The main strengths of this study were the use of whole tumor sections and automated image analysis for quantification of biomarkers. Additionally, biomarkers analysis were performed prospectively at the time of diagnosis; this may have reduced the bias associated with sample collection and evaluation. This study comprised a large number of pDCIS cases where a detailed analysis of biomarker expression and quantification was performed.

In conclusion, pDCIS is heterogeneous and like IBCA can be classified into distinct subtypes. Since DCIS is a direct precursor of IBCA, it can be inferred that there are distinct pathways to tumor progression, based on the molecular subtypes. Contrary to previous observation, we have shown that invasion is associated with significant increase in Ki67 index and decrease in ER, PR, and HER2 expression. Although most breast carcinomas maintain their phenotype during tumor progression, in some there is a change in phenotype possibly as a result of clonal evolution. Stromal factors may also play a role in influencing tumor growth and biomarker expression. 

## Figures and Tables

**Table 1:** Comparison of clinicopathologic parameters in pDCIS versus IBCA.

Variables	pDCIS (*n* = 118)	IBCA (*n* = 100)	*P* value*
Age (mean)	61.4 ± 1.03	61.8 ± 0.87	NS
Tumor size (mean)	2.95 ± 0.23	3.37 ± 0.32	NS
Tumor grade			
Low	9 (7.6%)	11 (11%)	NS
Intermediate	46 (38.9%)	49 (49%)	NS
High	63 (53.3%)	40 (40%)	0.0570

*Chi-square test.

NS: not significant.

**Table 2:** A comparison of biomarker expression in pDCIS versus IBCA.

Tumor biomarkers	pDCIS	IBCA	*P* value*
ER+	97 (82.2%)	66 (66%)	*P* = 0.0077
ER−	21 (17.7%)	34 (34%)	
PR+	81 (68.6%)	50 (50%)	*P* = 0.0057
PR−	37 (31.3%)	50 (50%)	
HER2+ (FISH)	36 (30.5%)	23 (23%)	*P* = 0.2859
HER2−	82 (69.4%)	74 (74%)	
Ki67 > 10%	78 (66.1%)	84 (84%)	*P* = 0.0030
Ki67 < 10%	40 (33.8%)	16 (16%)	
P53 >10%	27 (22.8%)	26 (26%)	*P* = 0.6363

P53 <10%	91 (77.1%)	74 (74%)
Total	118	100	

*Chi-square test; *P* < 0.05 is significant.

**Table 3:** The relationship of pDCIS grade with quantitative biomarker expression.

Variables	DCIS GRADE			*P* value (ANOVA)
No. of cases	Grade 1 (9)	Grade 2 (46)	Grade 3 (63)	

ER score (%)	89.7 ± 8.99	80.9 ± 4.78	58.6 ± 5.46	*P* = 0.0037
PR score (%)	62.0 ± 14.3	53.1 ± 5.42	31.9 ± 4.69	*P* = 0.0047
Ki67 (%)	12.7 ± 2.61	12.8 ± 1.38	25.6 ± 2.54	*P* = 0.0002
P53 (%)	10.0 ± 5.05	8.83 ± 3.11	21.0 ± 4.03	*P* = 0.0029
BCL-2 (%)	93.8 ± 6.25	75.7 ± 5.78	44.5 ± 6.78	*P* = 0.001

**Table 4:** Relationship of tumor grade with quantitative biomarker expression in IBCA.

Variables	IBCA grade			*P* value (ANOVA)
grade	Grade 1 (11)	Grade 2 (49)	Grade 3 (40)	

ER-score (%)	84.4 ± 8.80	75.3 ± 5.18	29.65 ± 6.69	*P* < 0.0001
PR-score (%)	49.1 ± 13.6	35.16 ± 5.34	14.30 ± 4.71	*P* = 0.0051
Ki67 (%)	14.7 ± 2.40	29.71 ± 3.55	61.2 ± 4.17	*P* < 0.0001
P53 (%)	11.8 ± 8.23	10.39 ± 3.26	27.95 ± 6.26	*P* = 0.0180

**Table 5:** The prevalence of the molecular subtypes in pDCIS versus IBCA by IHC.

Molecular subtypes	Pure DCIS	IBCA	*P* value
Luminal-A	44 (37.3%)	21 (21%)	0.0113
Luminal-B	31 (26.3%)	30 (30%)	0.5490
HER2*	36 (31%)	23 (23%)	0.2252
Triple negative	7 (6%)	26 (26%)	<0.0001

Total	118	100	

*Luminal-HER2 and HER2 subtypes are combined together as one group.

**Table 6:** A comparison of Ki67 indices and p53 overexpression in the different subtypes of pDCIS and IBCAs.

Tumor type	Luminal-A	Luminal-B	Lum-HER2	HER2	Triple negative	*P* value (ANOVA)
DCIS	44 (37.3%)	31 (26.3%)	22 (8.6%)	14 (11.8%)	7 (6%)	
Ki67 (%)	6.98 ± 0.52	23.8 ± 2.22	23.27 ± 2.72	33.57 ± 4.87	39.57 ± 13.6	*P* < 0.0001
P53 (%)	7.02 ± 2.89	12.3 ± 3.92	17.8 ± 6.10	24.4 ± 8.53	52.3 ± 18.6	*P* = 0.0014

IBCA	21 (21%)	30 (30%)	15 (15%)	8 (8%)	26 (26%)	
Ki67 (%)	7.05 ± 0.75	33.7 ± 2.53	41.4 ± 6.50	58.0 ± 9.46	67.5 ± 6.04	*P* < 0.0001
P53 (%)	2.15 ± 0.81	6.87 ± 2.12	36.3 ± 10.1	23.1 ± 15.1	31.0 ± 8.04	*P* < 0.0001

**Table 7:** Comparison of mean Ki67 indices in molecular subtypes of pDCIS and IBCA.

subtypes	Ki67	Ki67	*P* value
	DCIS	IBCA	

LUMINAL-A	6.98 ± 0.52 (*n* = 44)	7.05 ± 0.75 (*n* = 21)	*P* > 0.05
LUMINAL-B	23.8 ± 2.22 (*n* = 31)	33.76 ± 2.53 (*n* = 30)	*P* > 0.05
LUM-HER2	23.27 ± 2.72 (*n* = 22)	41.67 ± 6.50 (*n* = 15)	*P* < 0.01
HER2	33.57 ± 4.87 (*n* = 14)	58.00 ± 9.46 (*n* = 8)	*P* < 0.01
TRIPLE NEG	39.57 ± 13.64 (*n* = 7)	67.52 ± 6.04 (*n* = 26)	*P* < 0.01

*P value calculated by two-way ANOVA followed by Bonferroni post hoc test.
